# Efficacy and Safety of Oral Ibandronate versus Intravenous Zoledronic Acid on Bone Metabolism and Bone Mineral Density in Postmenopausal Japanese Women with Osteoporosis

**DOI:** 10.3390/jcm10225420

**Published:** 2021-11-20

**Authors:** Masashi Uehara, Yukio Nakamura, Takako Suzuki, Masaki Nakano, Jun Takahashi

**Affiliations:** 1 Department of Orthopaedic Surgery, Shinshu University School of Medicine, Matsumoto-City 390-8621, Nagano, Japan; masashi_u560613@yahoo.co.jp (M.U.); takako1119@shinshu-u.ac.jp (T.S.); masakin04@shinshu-u.ac.jp (M.N.); jtaka@shinshu-u.ac.jp (J.T.); 2 Department of Human Nutrition, Faculty of Human Nutrition, Tokyo Kasei Gakuin University, Chiyoda-ku 102-8341, Tokyo, Japan

**Keywords:** bone mineral density, efficacy, ibandronate, zoledronic acid, osteoporosis

## Abstract

There are no published clinical reports comparing ibandronate (IBN) treatment and zoledronic acid (ZOL) treatment in Japanese postmenopausal osteoporotic patients. This investigation therefore compared the efficacy and safety of the drugs on improving bone metabolism and bone mineral density (BMD) in Japanese postmenopausal women with primary osteoporosis. Eighty-two treatment-naïve primary osteoporotic female patients were randomly divided into IBN-treated or ZOL-treated groups. Bone turnover markers and BMD were examined immediately prior to treatment (baseline) and at 6, 12, 18, 24, and 30 months of therapy. Compared with baseline levels, the values of type 1 procollagen N-terminal propeptide, bone-specific alkaline phosphatase (BAP), urinary type-I collagen amino-terminal telopeptide (NTX), and tartrate-resistant acid phosphatase 5b were all significantly decreased at every time point in both groups apart from BAP at 30 months in the ZOL group, urinary NTX at 12 months in the ZOL group and at 24 and 30 months in both groups. Lumbar BMD values were significantly increased at 6, 12, 18, and 24 months in the IBN group and at 6 and 12 months in the ZOL group compared with pre-treatment levels. Hip BMD values were also significantly increased at 6, 12, 18, and 24 months in the IBN group and at 6, 12, and 18 months in the ZOL group compared with baseline values. The percentage changes of hip BMD at 18 and 24 months in the ZOL group were significantly higher than those in the IBN group (both *p* < 0.05). No remarkable adverse events were noted in either group. In conclusion, both IBN and ZOL significantly and safely improved bone turnover markers and BMD during 30 months of treatment in Japanese osteoporosis patients. The ZOL group tended to exhibit greater gains in BMD as compared with the IBN group, which merits further investigation.

## 1. Introduction

Including bisphosphonates (BPs) and the receptor activator of nuclear factor kappa-B ligand inhibitor denosumab, antiresorptive drugs are currently the most widely used osteoporosis medications. These agents increase bone mineral density (BMD) and reduce the risk of vertebral (40–70% reduction), non-vertebral (25–40% reduction), and hip (40–53% reduction) fractures in postmenopausal women with osteoporosis [1]. One intravenous BP, ibandronate (IBN), has recently been widely employed for the treatment of postmenopausal osteoporosis [2,3,4,5,6,7]. We and others have reported that IBN was well tolerated and associated with continued BMD gains and sustained bone turnover marker reductions in postmenopausal osteoporosis patients [2,3,4,5,6,7]. Oral IBN has been shown to be non-inferior to intravenous IBN [6]. In western countries, the efficacy of oral IBN has been demonstrated at a dose of 150 mg [2,3], while a dose of 100 mg is reportedly effective in Japan [4,6]. However, evidence on the merits of 100 mg oral IBN is lacking.

The once-yearly, third-generation, double nitrogen-containing, cyclic infusion BP zoledronic acid (ZOL) is frequently used for the treatment of postmenopausal as well as male osteoporosis [8,9]. However, there is no consensus on which drug is superior to improve bone metabolism in osteoporosis patients despite both medications routinely being prescribed in daily clinical practice.

This investigation compared the efficacy and safety of 100 mg oral IBN treatment and intravenous ZOL treatment for improving bone metabolism and BMD in Japanese postmenopausal osteoporosis patients.

## 2. Methods

### 2.1. Inclusion Criteria

The inclusion criteria were osteoporotic women of at least 20 years of age with at least 1 year of follow up.

### 2.2. Exclusion Criteria

Osteoporotic men, patients less than 20 years of age, and patients with less than 1 year of follow up were excluded from the study. Patients who had experienced a bone fracture less than 1 year prior to enrollment in this study were excluded as well.

### 2.3. Patients

**Figure 1:**
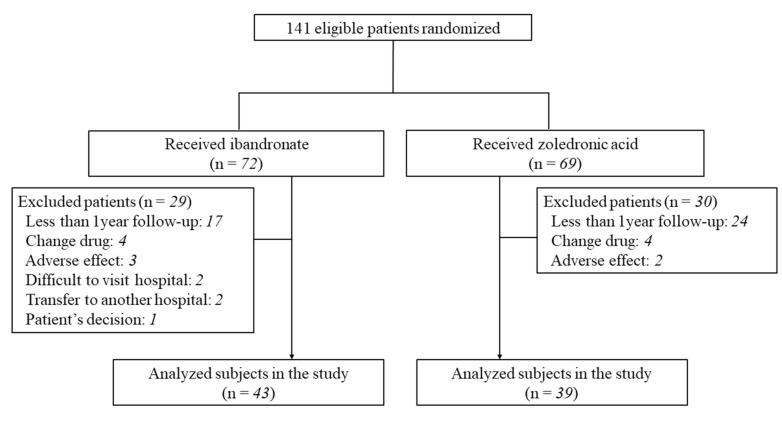
Participant flowchart of this study. A total of 141 patients were randomly divided into groups receiving either oral ibandronate or intravenous zoledronic acid. Eighty-two subjects were analyzed after 59 patients were excluded due to such reasons as insufficient follow up.

The participant flowchart of this study is shown in Figure 1. A total of 141 BP-treatment-naïve postmenopausal osteoporotic patients were prospectively recruited from our institutions between June 2017 and August 2019 for treatment with IBN or ZOL. After the randomization of the 141 patients into the group receiving oral IBN (IBN group) or the group treated with intravenous ZOL (ZOL group), 29 patients in the IBN group and 30 patients in the ZOL group were excluded. Eighty-two subjects were ultimately enrolled in the study after excluding 59 patients due to reasons including less than 1 year of follow up. The participants were randomly divided into the IBN group (43 women, mean ± standard deviation (SD) age: 70.9 ± 11.5 years) and the ZOL group (39 women, mean ± SD age: 67.8 ± 9.4 years). No patient had a history of medication that might have affected bone or calcium metabolism. The diagnosis of primary osteoporosis was made in accordance with the revised criteria established by the Japanese Society for Bone and Mineral Research [10]. In Japan, osteoporosis is defined as an average BMD value of the lumbar spine or femur in healthy young adults of less than 70% of the young adult mean [10]. In the IBN group, all subjects received 100 mg IBN orally every month. We confirmed, using patient interviews, that all subjects took the IBN tablets at least 1 h before eating or drinking. In the ZOL group, all subjects received 4 mg ZOL intravenously once a year. In the IBN group, 21 patients received vitamin D supplementation and 1 patient had calcium supplementation. Twenty-three patients in the ZOL group received vitamin D supplements.

### 2.4. Data Sources

We checked for BP use by means of patient self-reports. A history of fractures was ascertained by patient self-reports.

### 2.5. Clinic Sites

Clinic sites comprised university and municipal hospitals in Japan.

### 2.6. Randomization Methods

We used an enveloped method for randomization.

### 2.7. Adverse Effects Assessment

We assessed for adverse effects by patient self-reports at the time of consultation.

### 2.8. Evaluation and Statistical Analysis

Serum N-terminal propeptide of type 1 procollagen (PINP) and bone-specific alkaline phosphatase (BAP) were measured as bone formation markers using a chemiluminescent enzyme immunoassay and antibody radioimmunoassay, respectively. Serum tartrate-resistant acid phosphatase (TRACP)-5b and urinary N-terminal telopeptide of type-I collagen (NTX) were determined as markers of bone resorption using enzyme-linked immunosorbent assays. Serum whole parathyroid hormone (PTH) and 1-alpha, 25-dihydroxyvitamin D3 (1,25(OH)_2_D_3_) were assessed as additional bone turnover markers by immunoradiometric assays. Serum 25-hydroxyvitamin D (25OHD) was measured by a radioimmunoassay. Each marker value was determined just prior to IBN or ZOL administration (baseline) and at 6, 12, 18, 24, and 30 months of treatment. After overnight fasting, serum and first void urine samples were collected between 8:30 a.m. and 10:00 a.m. Immunoassays were performed by SRL, Inc. (Tokyo, Japan). Bone turnover marker scores were converted to a logarithmic scale since they were not normally distributed, while whole PTH, 1,25(OH)_2_D_3_, and 25OHD were analyzed using measured values. BMD was calculated using a dual-energy, X-ray absorption, fan-beam bone densitometer (Lunar Prodigy; GE Healthcare, Chicago, IL, USA) at the lumbar 1–4 levels of the spine (L-BMD) and bilateral total hips (H-BMD), before treatment and at 6, 12, 18, 24, and 30 months afterwards. H-BMD was calculated as the mean of both hips.

For both groups, we compared the changes in each parameter at each time point using paired *t*-tests and values between the group by means of Welch’s *t*-test. For all testing, a *p*-value of <0.05 was considered statistically significant.

This study was approved by the institutional ethical review board at Shinshu University School of Medicine (Nagano, Japan) prior to its commencement (No. 3700 and No. 3701) and by ClinicalTrials (NCT03183557 and NCT03186131). Written informed consent was obtained from all subjects at our institutions. This study was carried out in accordance with the approved guidelines. No financial support or equivalent was received for this investigation. The complete database of the cohort can be accessed at the Zenodo repository (doi:10.5281/zenodo.4295859).

## 3. Results

### 3.1. Patient Data

No significant differences in baseline patient characteristics were observed between the study groups (Table 1). A previous history of lower limb fracture was observed in two (4.7%) patients in the IBN group and seven (17.9%) patients in the ZOL group, although this difference was not significant (*p* = 0.08).

### 3.2. Levels of Serum Albumin-Corrected Calcium, Phosphorus, Whole PTH, 1,25(OH)_2_D_3_, and 25OHD

**Figure 2:**
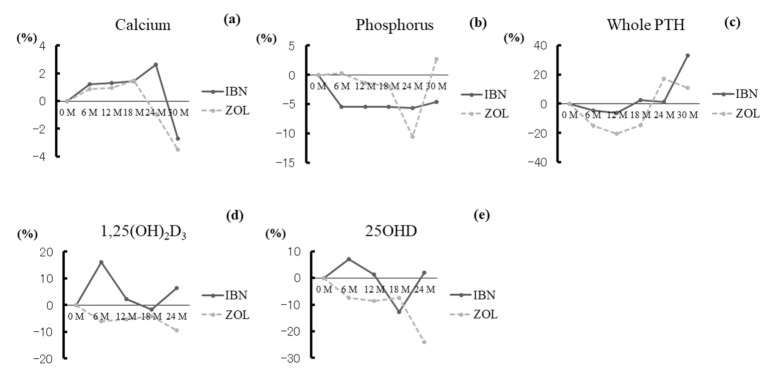
Percentage changes of serum albumin-corrected calcium (**a**), phosphorus (**b**), whole PTH (**c**), 1,25(OH)_2_D_3_ (**d**), and 25OHD (**e**). (**a**–**e**) Serum albumin-corrected calcium, phosphorus, whole PTH, 1,25(OH)_2_D_3_, and 25OHD levels did not change significantly versus baseline values in either the IBN group or the ZOL group at any time point.

We observed no remarkable differences between the groups for any marker. The percent changes of serum albumin-corrected calcium, phosphorus, whole PTH, 1,25(OH)_2_D_3_, and 25OHD did not change significantly versus baseline values in either the IBN group or the ZOL group at any time point (Figure 2a–e).

### 3.3. Bone Turnover Markers

#### 3.3.1. Markers of Bone Formation

**Figure 3:**
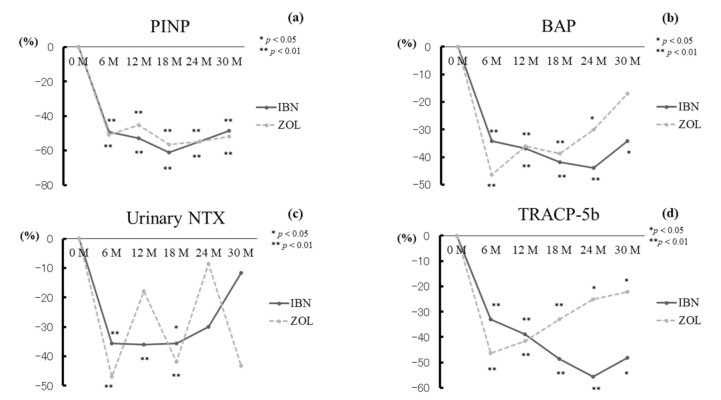
Percentage changes of PINP (**a**), BAP (**b**), urinary NTX (**c**), and TRACP-5b (**d**). All values were significantly decreased from baseline values at every time point in both groups apart from BAP at 30 months in the ZOL group and urinary NTX at 12 months in the ZOL group and at 24 and 30 months in both groups. *: *p* < 0.05, **: *p* < 0.01.

There were no significant differences between the groups at any time point for PINP. PINP values were significantly decreased from pre-treatment levels at 6–30 months in both groups (all *p* < 0.01) (Figure 3a).

We observed no significant differences between the groups at any time point for BAP. BAP levels were significantly decreased from baseline at 6, 12, 18, and 24 months (all *p* < 0.01) and at 30 months (*p* < 0.05) in the IBN group, and at 6, 12, and 18 months (all *p* < 0.01) and at 24 months (*p* < 0.05) in the ZOL group (Figure 3b).

#### 3.3.2. Markers of Bone Resorption

There were no significant differences between the groups at any time point for urinary NTX. Urinary NTX values were significantly decreased from baseline at 6 and 12 months (both *p* < 0.01) and at 18 months (*p* < 0.05) in the IBN group and at 6 and 18 months (both *p* < 0.01) in the ZOL group (Figure 3c).

We detected no significant differences between the groups at any time point for TRACP-5b. TRACP-5b levels were significantly decreased at 6–24 months (all *p* < 0.01) and at 30 months (*p* < 0.05) in the IBN group and at 6–18 months (all *p* < 0.01) and at 24 and 30 months (both *p* < 0.05) in the ZOL group (Figure 3d).

### 3.4. L-BMD and H-BMD

**Figure 4:**
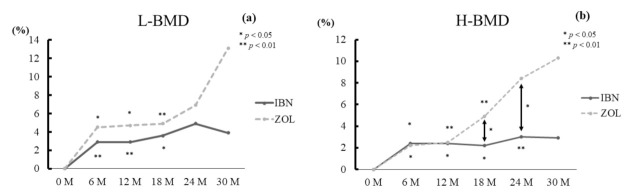
Percentage changes of L-BMD (**a**) and H-BMD (**b**). The ZOL group tended to exhibit enhanced gains in L-BMD and H-BMD compared with the IBN group. However, these differences were not significant except for the percentage changes of H-BMD at 18 and 24 months. * *p* < 0.05, ** *p* < 0.01.

There were no significant differences between the groups for L-BMD at any time point. The value of L-BMD was significantly increased at 6, 12, and 24 months (all *p* < 0.01) and at 18 months (*p* < 0.05) in the IBN group and at 6 and 12 months (both *p* < 0.05) in the ZOL group over pre-treatment levels (Figure 4a).

The percent changes of H-BMD at 18 and 24 months in the ZOL group were significantly higher than those in the IBN group (both *p* < 0.05). The value of H-BMD was significantly increased at 6, 12, and 18 months (all *p* < 0.05) and at 24 months (*p* < 0.01) in the IBN group and at 6 months (*p* < 0.05) and at 12 and 18 months (both *p* < 0.01) in the ZOL group versus the baseline values. (Figure 4b).

### 3.5. Safety Evaluations

Nausea was observed after commencement in one patient in the ZOL group, with no subsequent adverse effects. No serum calcium level abnormalities or fractures were observed in either group during the observational period. The main reasons for drug withdrawal in this study were changes due to poor treatment efficacy or at the patient’s request from side effects. The reasons for withdrawal were unknown for 17 patients in the IBN group and 24 patients in the ZOL group, who were followed for less than 1 year.

## 4. Discussion

The present randomized study compared the efficacy and safety of two commonly prescribed osteoporosis drugs, oral IBN and intravenous ZOL, in Japanese postmenopausal osteoporosis patients. During the 30-month follow-up period, both study groups exhibited significant and comparable changes in bone turnover markers and increases in BMD without major adverse effects. Although not significantly, except for the percentage changes of H-BMD at 18 and 24 months, the patients treated with ZOL tended to exhibit greater gains in BMD.

Several reports have described the effects of oral BPs for osteoporosis management [11,12,13]. A comparison of oral and intravenous BPs revealed comparable effects on changes in BMD and bone turnover markers [14]. Hagino et al. found that oral IBN produced BMD gains similar to those produced by monthly intravenous IBN [11]. In Korean osteoporosis patients, monthly oral IBN provided better anti-fracture efficacy than monthly oral risedronate [12]. However, patient adherence to some osteoporosis regimens is often unsatisfactory. Kishimoto et al. examined compliance and persistence with BPs for osteoporosis and concluded that monthly regimens had significantly better adherence than weekly and daily ones [13]. The above findings indicate that monthly oral IBN formulations are preferable in terms of efficacy, safety, and adherence. In Japan, the effectiveness of oral IBN has mainly been demonstrated at doses of 100 mg, although evidence remains insufficient.

A recent study showed that intravenous ZOL provided early pain relief and complete reversal of transient hip osteoporosis [15]. Anastasilakis et al. found that a single intravenous ZOL treatment prevented bone loss for at least 2 years, independently of bone turnover rate [16]. On the other hand, ZOL was also considered to cause acute-phase reactions (APRs), although non-steroidal anti-inflammatory drugs could reduce such effects [17]. In the present trial, no APRs were observed during the observational period.

BPs cannot be used in patients with severe renal dysfunction [18]. Although Green et al. demonstrated the drug’s higher antiresorptive capacity and relatively low renal toxicity in thyroparathyroidectomized rats, many cases of ZOL-associated renal toxicity or failure in relation to dosage have been reported [19]. For this reason, patients receiving ZOL should be monitored closely for potential deterioration in renal function. In the present study, no adverse events or renal failure were reported.

In a previous report, IBN and ZOL were equivalent in suppressing bone resorption markers and improving pain in cancer patients [20,21]. A recent study evaluating pain and quality of life in postmenopausal osteoporosis patients treated with denosumab, IBN, or ZOL also revealed no significant differences in any of the measured pain parameters or quality of life domains [22]. In this study, oral IBN and intravenous ZOL appeared comparably effective in improving bone turnover markers and BMD, and so no conclusion could be drawn as to which drug would be better to start with. The choice of prescription may therefore be based on the administration method acceptable to the patient and the prescribing physician’s experience of use.

The main limitations of this investigation are a relatively small size and short-term observational period. Another limitation was that the main outcomes of the study focused on BMD and not fractures. In addition, it is unclear whether our findings can be generalized to other groups apart from elderly Japanese women. The number of patients who were followed after 24 months was small as compared with the initial number of patients; the high number of dropouts was possibly attributable to the prospective nature of the study. Lastly, serum CTX is reportedly superior to urine NTX for assessing bone resorption, although we have demonstrated the reliability of urine NTX in previous reports [23,24]. Further trials are required to validate our results with respect to increasing BMD values and fracture prevention by oral IBN or intravenous ZOL.

## 5. Conclusions

This study showed comparable results for 100 mg of oral IBN and 4 mg of intravenous ZOL on significantly and safely improving bone turnover markers and BMD during 30 months of treatment in Japanese postmenopausal osteoporosis patients. The ZOL group tended to exhibit enhanced gains in L-BMD and H-BMD as compared with the IBN group. However, these differences were not significant, a except for the percentage changes of H-BMD at 18 and 24 months, and both groups displayed similarly ameliorated bone turnover markers. Larger studies are warranted for the potential differences in BMD gains and fracture prevention between the drugs.

## Figures and Tables

**Table 1:** Baseline patient characteristics.

Characteristic	IBN Group (*n* = 43)	ZOL Group (*n* = 39)	*p*-Value
Age (years)	70.9 ± 11.5	67.8 ± 9.4	0.22
BMI (kg/m^2^)	21.9 ± 3.6	22.1 ± 3.7	0.77
Previous history of lower limb fracture (*n*)	2	7	0.08
Previous history of lower limb fracture (SMD)	0.000055 ± 1.0	0.000018 ± 1.0	1.00
Previous history of upper limb fracture (*n*)	3	1	0.62
Previous history of vertebral fracture (*n*)	12	7	0.31
Previous history of pelvic fracture (*n*)	2	1	1.00
Serum albumin-corrected calcium (mg/dL)	9.2 ± 0.9	9.3 ± 0.5	0.21
Serum phosphorus (mg/dL)	3.8 ± 0.5	3.7 ± 0.5	0.18
Serum BAP (μg/L)	14.8 ± 8.4	17.3 ± 10.5	0.13
Serum PINP (ng/mL)	61.1 ± 42.7	67.7 ± 45.5	0.54
Serum TRACP-5b (mU/dL)	465 ± 212	552 ± 273	0.12
Urinary NTX (nmol BCE/mmoL CRE)	61.2 ± 57.8	50.9 ± 31.3	0.24
Serum whole PTH (pg/mL)	29.4 ± 15.1	32.0 ± 12.5	0.40
Serum 1,25(OH)_2_D_3_ (pg/mL)	53.4 ± 18.8	60.5 ± 24.8	0.16
Serum 25OHD (ng/mL)	15.6 ± 6.3	16.2 ± 5.0	0.66
eGFR (mL/min/1.73 m^2^)	62.4 ± 20.6	69.8 ± 12.5	0.07
Lumbar BMD (g/cm^2^)	0.89 ± 0.18	0.87 ± 0.23	0.70
T score	−1.64 ± 1.27	−1.87 ± 1.68	0.38
Total hip BMD (g/cm^2^)	0.69 ± 0.13	0.69 ± 0.16	0.91
T score	−1.98 ± 1.08	−1.79 ± 1.26	0.35

BMI: body mass index, SMD: standardized mean difference, BAP: bone-specific alkaline phosphatase, PINP: N-terminal propeptide of type 1 procollagen, TRACP-5b: tartrate-resistant acid phosphatase 5b, NTX: N-terminal telopeptide of type-I collagen, PTH: parathyroid hormone, 1,25(OH)_2_D_3_: 1-alpha, 25-dihydroxyvitamin D3, 25OHD: 25-dihydroxyvitamin D, eGFR: estimated glomerular filtration rate, BMD: bone mineral density.

## Data Availability

The complete database of the cohort can be accessed at the Zenodo repository (doi:10.5281/zenodo.4295859).
